# Spectroscopic study of Gd nanostructures quantum confined in Fe corrals

**DOI:** 10.1038/srep12092

**Published:** 2015-07-10

**Authors:** R. X. Cao, L. Sun, B. F. Miao, Q. L. Li, C. Zheng, D. Wu, B. You, W. Zhang, P. Han, S. D. Bader, W. Y. Zhang, H. F. Ding

**Affiliations:** 1 National Laboratory of Solid State Microstructures and Department of Physics, Nanjing University, 22 Hankou Road, Nanjing 210093, People’s Republic of China; 2 Collaborative Innovation Center of Advanced Microstructures, Nanjing University, 22 Hankou Road, Nanjing 210093, P. R. China; 3 School of Electronic Science and Engineering, Nanjing University, 22 Hankou Road, Nanjing 210093, People’s Republic of China; 4 Materials Science Division, Argonne National Laboratory, Argonne, Illinois 60439, USA

## Abstract

Low dimensional nanostructures have attracted attention due to their rich physical properties and potential applications. The essential factor for their functionality is their electronic properties, which can be modified by quantum confinement. Here the electronic states of Gd atom trapped in open Fe corrals on Ag(111) were studied via scanning tunneling spectroscopy. A single spectroscopic peak above the Fermi level is observed after Gd adatoms are trapped inside Fe corrals, while two peaks appear in empty corrals. The single peak position is close to the higher energy peak of the empty corrals. These findings, attributed to quantum confinement of the corrals and Gd structures trapped inside, are supported by tight-binding calculations. This demonstrates and provides insights into atom trapping in open corrals of various diameters, giving an alternative approach to modify the properties of nano-objects.

Electronic structure is the fundamental property of the material and of great importance for their applications. Low dimensionality, the surface enhancement and quantum size effects etc., make the properties of nanostructures significantly different from that of bulk^1^,^2^,^3^,^4^,^5^,^6^, generating many new and fascinating magnetic^7^,^8^,^9^,^10^,^11^,^12^, mechanical^13^,^14^,^15^, optical^16^,^17^ and superconducting^18^,^19^,^20^ properties. This enriches the material selection and new functionality design, casting strong potential in applications such as information storage^11^,^21^ and transfer^10^,^22^. Scanning tunneling microscopy (STM)^23^ and spectroscopy (STS)^24^,^25^ are powerful tools to investigate electronic properties of nanostructures and topographic information simultaneously with atomic-level spatial resolution. Previous findings via STM and STS have found that the electronic states can be modified by quantum confinement perpendicular to the surface^26^,^27^ and on the surface^2^,^28^,^29^. Moreover, quantum confinement can influence atom diffusion^30^,^31^,^32^ and suppress the statistical fluctuations in the number of atoms within a nanostructure^33^, resulting in fabrication with atomic-level precision. Therefore, exploring the electronic properties in this atomic structure will be interesting and meaningful for the atomic-level nanomaterial design.

Herein we report on an experimental investigation of the electronic states of Gd adatoms trapped in open Fe corrals of varied size^33^ as regulated by quantum confinement. STS results near the Fermi level show two characteristic spectroscopic peaks in empty corrals with the peak positions shift toward lower energy with increasing corral diameter. Once a corral traps enough Gd adatoms the higher energy peak remains, while lower energy peak disappears. These experimental findings are attributed to the quantum confinement of the corrals and the subsequently trapped Gd structures as is supported by tight-binding (TB) calculations of the local density of states (LDOS).

## Results

**Figure 1:**
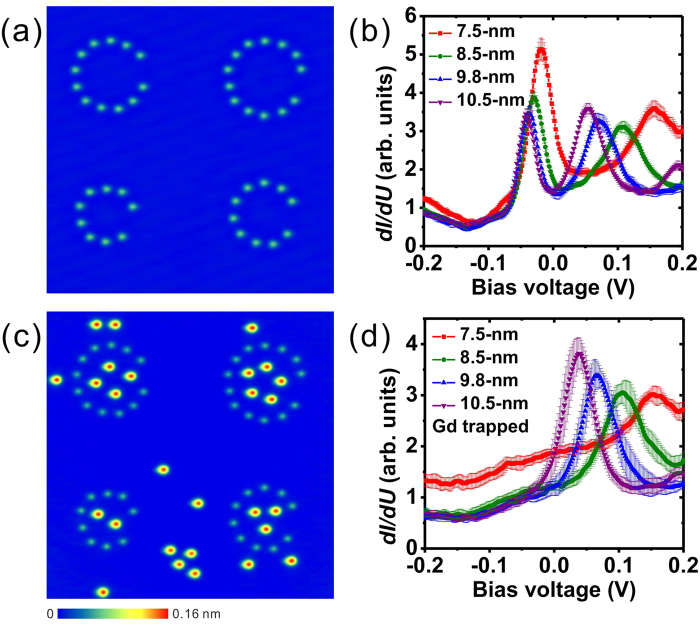
The changing of electronic properties after atom trapping. STM image of an array of open Fe corrals of diameters 7.5, 8.5, 9.8 and 10.5 nm before (**a**) and after (**c**) trapping Gd adatoms. The image sizes of (**a**) and (**c**) are 40 × 40 nm^2^. The *dI*/*dU* spectra are measured at the centers of different corrals before (**b**) and after (**d**) trapping Gd adatoms.

Fe and Gd adatoms are deposited onto Ag(111) and the Fe nanocorrals are built via atom manipulation. As demonstrated previously^33^, stable open nanocorrals on the Ag(111) surface can be built with Fe adatoms that have a favorable diffusion barrier. Figure 1(a) shows the STM image of a 2 × 2 array of open Fe corrals with four different diameters (7.5, 8.5, 9.8 and 10.5 nm) on Ag(111). The image size is 40 × 40 nm^2^. Two neighboring corrals are separated by 20 nm. In order to study the influence on the electronic properties due to Gd atomic trapping, we first performed spectroscopy at the center of each corral as a reference. The spectroscopy was measured for more than ten times. The average curve and statistical error were plotted in Fig. 1(b). Two characteristic peaks are observed that shift to lower energy with increasing corral diameter. We note that Gd adatoms are driven out of the scanning area with this scanning condition (−0.5 V, 1 nA)^33^.

Moreover, when a different condition (0.5 V, 2 pA) is used for the scanning, Gd adatoms can diffuse back to the scanning area and be trapped by the open corrals, driven by a surface-state-mediated, long-range interaction^33^. The opening in the corral forms a gate to regulate the flow of Gd atoms traveling in and out, resulting in quantized atom trapping and a suppression of statistical fluctuations, as demonstrated in Fig. 1(c). Four open corrals of different-size trap 2, 3, 4 and 5 Gd adatoms, respectively. Thus, by changing the diameter of the open corrals, this approach offers the ability to modify the properties of nano-objects. STS provides a powerful tool to investigate the LDOS of these nanosturctures. The LDOS taken at the centers of the Gd-filled corrals are shown in Fig. 1(d). Comparing with the results before trapping Gd, we find that there is only one spectral peak, the other one at lower energy disappears.

**Figure 2:**
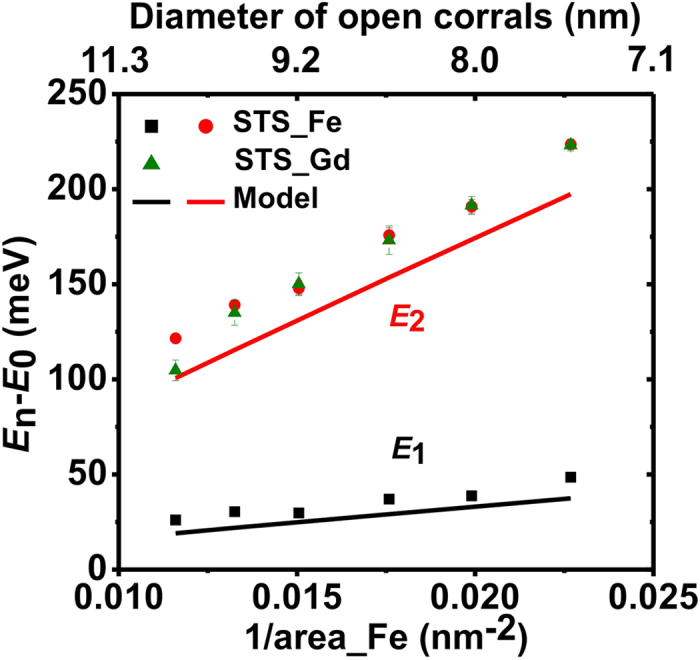
Comparison with confinement model. Comparison of the experimental (black rectangles and red circles are for empty Fe corrals, green triangles are for Gd-filled corrals) and confinement-model-derived (black and red lines) positions of STS peaks in different-sized corrals.

The same measurement was performed in corrals with two additional sizes (8.0- and 9.2-nm diameter) and their spectroscopic peak positions are plotted in Fig. 2, together with the data shown in Fig. 1. The peaks at lower energy (black rectangles) disappear after the trapping of the Gd adatoms. The peak positions in the Gd-filled corrals (green triangles) are close to the higher-energy spectral peak of the empty corrals (red circles). These electronic properties can be qualitatively understood by the confinement model discussed previously^2^,^3^. Lateral confinement of the surface-state electrons will cause a series of discrete energy levels corresponding to the two-dimensional particle-in-a-box eigenstates. For a circular corral with continuous hard wall barriers, the energy levels can be expressed as:


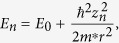


where *E*_0_ is the onset energy of −67 meV, m^*^ is the effective mass of 0.42 *m*_*e*_ of the Ag(111) surface state^34^, *r* is the radius of the corral, and *z*_*n*_ is the *n*^th^ solution of the zeroth order Bessel function *J*_0_(z). Utilizing this model, the energy levels are plotted as a function of inverse corral area in Fig. 2, labeled *E*_1_ (black line) and *E*_2_ (red line). The quantum-confinement model in general agrees with the experimental findings in the empty Fe corrals. A slight quantitative deviation, especially at higher energy, can be observed, as had been also reported by Crommie *et al.*^2^ This deviation could be attributed to a reduced confinement effect induced by the discrete corral wall and the finite wall barrier with specific phase shift, which are different from the simple hard-wall model above. Moreover, we compared the experimental STS peaks in the Gd-filled corrals with the confinement model (see Supplementary Fig. S1). The qualitative agreement illustrates that the spectroscopic peaks are due to the quantum confinement.

**Figure 3:**
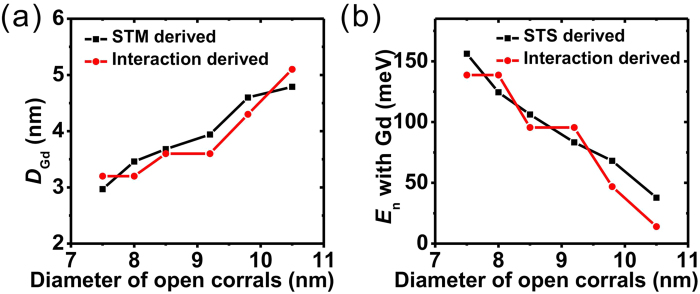
The origin of the spectroscopy. (**a**) The diameters of the circular area formed by Gd adatoms, which are derived from STM images (black rectangles) and long-range interactions including total energy minimization (red circles). (**b**) The spectroscopic peak positions derived from STS measurement (black rectangles) and confinement model concerning interaction-determined Gd locations (red circles).

In the following, the origin of the spectroscopy in the Gd-filled corrals is explored. Why are they so close to the higher energy peak of the empty Fe corrals? Since the electronic properties arise from the quantum confinement of the Gd nanostructures, the location of the Gd adatoms are of paramount importance. The Gd locations are mainly determined by the distribution of the long-range interaction between the Gd adatoms themselves, and their interaction with the Fe corral^32^,^33^,^35^. We arranged the Gd adatoms symmetrically in a circular orbit in the Fe corrals and calculated the total interactions as the function of the orbit diameter by summing up the long-range interaction energies for every pair of Gd-Gd adatoms and Gd-Fe adatoms^32^. Then the optimal Gd locations were found via total energy minimization. The interaction-determined diameters of the Gd adatoms are plotted (red circles) in Fig. 3(a), comparing with the diameters derived from the STM images (black rectangles). The calculation only includes two-body interactions, however, the agreement illustrates that the Gd structure is driven by the long-range interaction. The energy levels of the Gd structures are plotted [red circles in Fig. 3(b)] according to the calculated Gd locations and the confinement model. The calculated peak positions are close to those of the STS measurements. This demonstrates that the spectral change is caused by the quantum confinement of the Gd structures as determined by the surface-state-mediated, long-range interaction.

**Figure 4:**
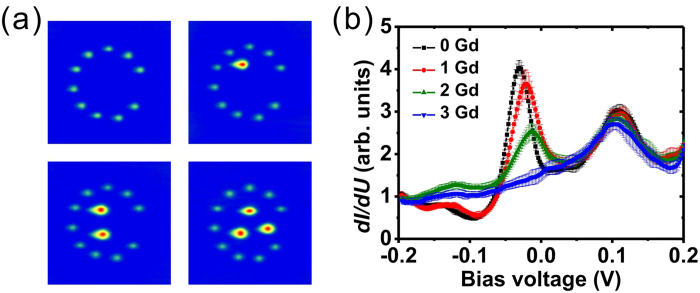
The step-by-step evolution of spectroscopy. (**a**) STM images of the 8.5-nm open Fe corral with 0, 1, 2 and 3 Gd adatoms trapped inside. The image sizes are 15 × 15 nm^2^. (**b**) The evolution of the STS at the center of the corral during the trapping of Gd adatoms.

Knowing the reason for the change of electronic states after the trapping of the Gd adatoms, it is interesting to investigate the step-by-step evolution of spectroscopy as the corral traps each Gd adatom. The empty Fe corral of diameter 8.5 nm was scanned and the diffusion of Gd adatoms was tracked at 4.2 K. Once one Gd adatom is trapped inside the corral, the sample is cooled to 3.0 K to avoid more Gd trapping, and the measurement is performed. Then the sample is warmed to 4.2 K to trap another Gd adatom. Finally, the topographic [Fig. 4(a)] and spectroscopic [Fig. 4(b)] information for the 8.5-nm corral with 0, 1, 2 and 3 trapped Gd adatoms was obtained. The evolution of the spectroscopy in the corral can be clearly traced: the intensity of the peak at lower energy decreases to zero gradually as its position shifts slightly toward higher energy; the intensity and position of the peak at higher energy are nearly the same. This can be understood as follows. When more Gd adatoms are trapped inside the corral, the electronic contribution from the corral starts to be screened by the new nanostructure formed by Gd adatoms. Interestingly, the spectroscopy of the Gd structure has its characteristic peak near the higher energy peak in the empty corral. Combining these two effects, the evolution of the STS with increasing number of trapped Gd adatoms can be explained. We note that the difference around −0.1 V came from the changing tip condition, as shown by the spectroscopy of the pure Ag(111) surface without nearby adatoms. This tendency was also qualitatively reproduced by the TB calculation (Supplementary Fig. S2).

This spectral change can also be understood qualitatively as follows. There are two different electronic states in experimental energy range for each empty corral. The standing wave of the second state has a node close to the position of Gd adatoms driven by the long-range interaction minimum (see Supplementary Fig. S3). Therefore, the second state can survive with the new Gd boundary. However, the first state without a node at the Gd positions is massively affected.

**Figure 5:**
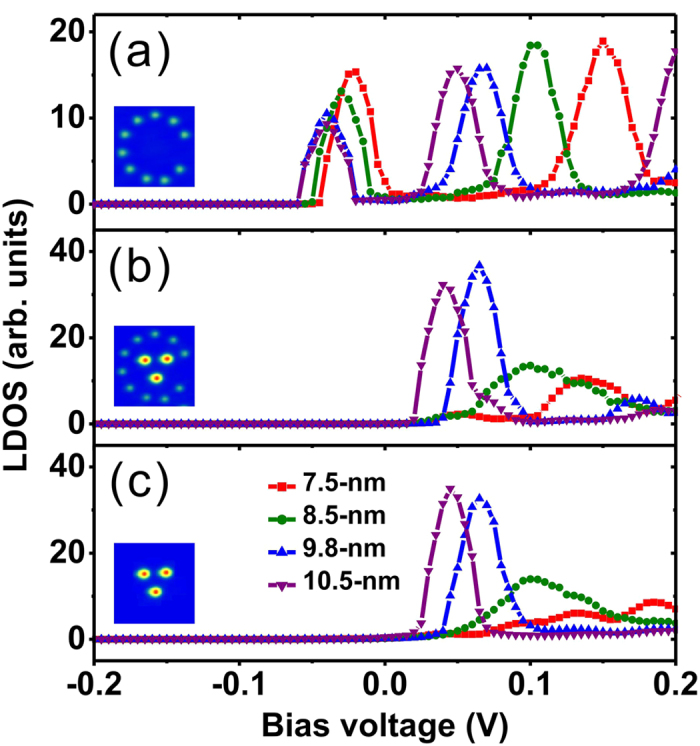
TB calculated LDOS. The results were calculated at the centers of corrals of different sizes: (**a**) for empty corrals, (**b**) for Gd occupied corrals, and (**c**) for Gd structure after artificially removing Fe corrals. The insets show corresponding STM images for 8.5-nm corral.

To obtained quantitative agreement, TB calculations were also performed for corrals of different sizes. The curves in Fig. 5(a) show the calculated LDOS at the centers of four different corrals. The adatom positions are the same as in the experiments. The two spectral peaks and their shifting towards lower energy in larger corrals are reproduced. After trapping the Gd adatoms, the calculated LDOS only has the higher energy peak [Fig. 5(b)], in agreement with experiment. In order to show that the spectroscopic properties of the Gd-filled corrals mainly come from the Gd structures, the Fe corrals were artificially removed from the calculation. This spectroscopy of the artificial structure shown in the inset of Fig. 5(c) stays nearly the same except in the 7.5-nm corral, as described below.

**Figure 6:**
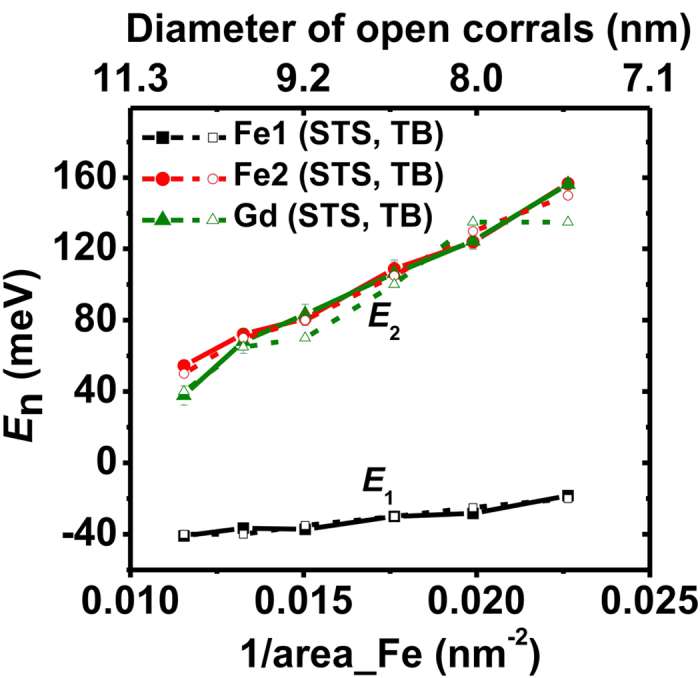
Comparison of the experimental and TB calculated results. The experimental spectroscopic peak positions are shown as solid symbols and TB calculated LDOS peak positions are shown as open symbols. Black and red symbols represent the peak positions at lower and higher energies in empty Fe corrals, respectively. Green symbols represent the peak positions for the Gd-filled corrals.

The peak positions derived from STS measurements (solid symbols) and TB calculations (open symbols) are compared in Fig. 6. In empty Fe corrals, the calculated peak positions agree with the experimental results both at lower (black rectangles) and higher energies (red circles). After Gd adatoms are trapped in the corrals, the agreement still exists except for the 7.5-nm corral (green triangles). This deviation may come from the reduced confinement effect as only two Gd atoms are involved, or from the modification of the TB Hamiltonian when the size of the Gd structure shrinks.

In summary, the electronic structures in Gd nanostructures trapped in open Fe corrals were experimentally investigated. Two characteristic spectroscopic peaks are observed near the Fermi level in empty corrals, and they shift toward lower energies with increasing corral diameter. After the trapping of Gd adatoms, there is only one peak, whose position is close to that of the higher energy peak in the empty corral. These findings are explained by the quantum confinement of the corrals and the Gd structures inside, as determined by a surface-state-mediated, long-range interaction. TB calculations were performed that are in quantitative agreement with the experimental results. These findings demonstrate that by changing the diameter of the open corrals atomic structures with atomic-level precision can be achieved with tailored electronic structures.

## Methods

### Experimental techniques

The experiments were performed in an ultrahigh vacuum chamber (2 × 10^−11^ mbar) equipped with a low-temperature STM and a sputter gun. The preparation of the single-crystal Ag(111) substrate and subsequent Fe and Gd *in-situ* depositions have been described previously^32^,^33^. For imaging Gd adatoms, the sample can be cooled to ~3 K by pumping on liquid He. The scanning conditions for Gd imaging were: bias voltage *U* = 0.5 V, and tunneling current *I*_*t*_ = 2 pA. The bias voltage refers to the sample voltage with respect to the tip. The W tip was flashed *in-situ* with an *e*-beam heating device to remove the oxide layer^36^. After cleaning, reproducible spectroscopy data are readily obtained. Spectroscopy measurements were performed via the modulation technique utilizing a 10-mV amplitude and 6.09-kHz frequency.

### Method of calculation

To understand the experimental results, TB calculations were performed as described previously^37^,^38^. Periodic boundary conditions are used with a unit cell between 55 × 55 and 65 × 65 (number of Ag atoms between the centers of two neighboring corrals) according to the different sizes of the Fe corrals. Thus, each corral can be considered as being isolated. The position of the adatoms is determined experimentally and their effect on the Hamiltonian is attributed to a modification of the on-site potential of the nearby Ag(111) lattice. Then the LDOS at the center of the corral is calculated.

## Additional Information

**How to cite this article**: Cao, R. X. *et al.* Spectroscopic study of Gd nanostructures quantum confined in Fe corrals. *Sci. Rep.* **5**, 12092; doi: 10.1038/srep12092 (2015).

## Supplementary Material

Supplementary Information

## References

[b1] Crommie M. F., Lutz C. P. & Eigler D. M. Imaging standing waves in a two-dimensional electron gas. Nature 363, 524 (1993).

[b2] Crommie M. F., Lutz C. P. & Eigler D. M. Confinement of electrons to quantum corrals on a metal surface. Science 262, 218 (1993). 17841867 10.1126/science.262.5131.218

[b3] Li J., Schneider W.-D., Berndt R. & Crampin S. Electron confinement to nanoscale Ag islands on Ag(111): A quantitative study. Phys. Rev. Lett. 80, 3332–3335 (1998).

[b4] Manoharan H. C., Lutz C. P. & Eigler D. M. Quantum mirages formed by coherent projection of electronic structure. Nature 403, 512–515 (2000). 10676952 10.1038/35000508

[b5] Nilius N., Wallis T. M. & Ho W. Development of one-dimensional band structure in artificial gold chains. Science 297, 1853 (2002). 12193641 10.1126/science.1075242

[b6] Gomes K. K., Mar W., Ko W., Guinea F. & Manoharan H. C. Designer Dirac fermions and topological phases in molecular graphene. Nature 483, 306–310 (2012). 22422264 10.1038/nature10941

[b7] Qiu Z. Q., Pearson J., Berger A. & Bader S. D. Short-period oscillations in the interlayer magnetic coupling of wedged Fe(100)/Mo(100)/Fe(100) grown on Mo(100) by molecular-beam epitaxy. Phys. Rev. Lett. 68, 1398–1401 (1992). 10046156 10.1103/PhysRevLett.68.1398

[b8] Li J., Przybylski M., Yildiz F., Ma X. D. & Wu Y. Z. Oscillatory magnetic anisotropy originating from quantum well states in Fe films. Phys. Rev. Lett. 102, 207206 (2009). 19519071 10.1103/PhysRevLett.102.207206

[b9] Oka H. *et al.* Spin-dependent quantum interference within a single magnetic nanostructure. Science 327, 843–846 (2010). 20150496 10.1126/science.1183224

[b10] Khajetoorians A. A., Wiebe J., Chilian B. & Wiesendanger R. Realizing all-spin–based logic operations atom by atom. Science 332, 1062–1064 (2011). 21551029 10.1126/science.1201725

[b11] Loth S., Baumann S., Lutz C. P., Eigler D. M. & Heinrich A. J. Bistability in atomic-scale antiferromagnets. Science 335, 196–199 (2012). 22246771 10.1126/science.1214131

[b12] Sun L. *et al.* Creating an artificial two-dimensional skyrmion crystal by nanopatterning. Phys. Rev. Lett. 110, 167201 (2013). 23679635 10.1103/PhysRevLett.110.167201

[b13] Tombler T. W. *et al.* Reversible electromechanical characteristics of carbon nanotubes under local-probe manipulation. Nature 405, 769–772 (2000). 10866192 10.1038/35015519

[b14] Yu M.-F. *et al.* Strength and breaking mechanism of multiwalled carbon nanotubes under tensile load. Science 287, 637–640 (2000). 10649994 10.1126/science.287.5453.637

[b15] Lee C., Wei X., Kysar J. W. & Hone J. Measurement of the elastic properties and intrinsic strength of monolayer graphene. Science 321, 385–388 (2008). 18635798 10.1126/science.1157996

[b16] Hache F., Ricard D. & Flytzanis C. Optical nonlinearities of small metal particles: Surface-mediated resonance and quantum size effects. J. Opt. Soc. Am. B 3, 1647, (1986).

[b17] Yin X. *et al.* Edge nonlinear optics on a MoS_2_ atomic monolayer. Science 344, 488–490 (2014). 24786072 10.1126/science.1250564

[b18] Guo Y. *et al.* Superconductivity modulated by quantum size effects. Science 306, 1915–1917 (2004). 15591197 10.1126/science.1105130

[b19] Brun C. *et al.* Reduction of the superconducting gap of ultrathin Pb islands grown on Si(111). Phys. Rev. Lett. 102, 207002 (2009). 19519063 10.1103/PhysRevLett.102.207002

[b20] Qin S., Kim J., Niu Q. & Shih C.-K. Superconductivity at the two-dimensional limit. Science 324, 1314–1317 (2009). 19407146 10.1126/science.1170775

[b21] Gambardella P. *et al.* Ferromagnetism in one-dimensional monatomic metal chains. Nature 416, 301–304 (2002). 11907571 10.1038/416301a

[b22] Menzel M. *et al.* Information transfer by vector spin chirality in finite magnetic chains. Phys. Rev. Lett. 108, 197204 (2012). 23003082 10.1103/PhysRevLett.108.197204

[b23] Binnig G., Rohrer H., Gerber C. & Weibel E. Tunneling through a controllable vacuum gap. Appl. Phys. Lett. 40, 178–180 (1982).

[b24] Hamers R. J., Tromp R. M. & Demuth J. E. Surface electronic structure of Si(111)-(7*7) resolved in real space. Phys. Rev. Lett. 56, 1972–1975 (1986). 10032824 10.1103/PhysRevLett.56.1972

[b25] Kaiser W. J. & Jaklevic R. C. Spectroscopy of electronic states of metals with a scanning tunneling microscope. IBM J. Res. Dev. 30, 411–416 (1986).

[b26] Altfeder I. B., Matveev K. A. & Chen D. M. Electron fringes on a quantum wedge. Phys. Rev. Lett. 78, 2815–2818 (1997).

[b27] Yang M. C. *et al.* Phase contribution of image potential on empty quantum well states in Pb islands on the Cu(111) surface. Phys. Rev. Lett. 102, 196102 (2009). 19518977 10.1103/PhysRevLett.102.196102

[b28] Pennec Y. *et al.* Supramolecular gratings for tuneable confinement of electrons on metal surfaces. Nat. Nanotech. 2, 99–103 (2007). 10.1038/nnano.2006.212 18654227

[b29] Kliewer J., Berndt R. & Crampin S. Controlled modification of individual adsorbate electronic structure. Phys. Rev. Lett. 85, 4936–4939 (2000). 11102155 10.1103/PhysRevLett.85.4936

[b30] Stepanyuk V. S., Negulyaev N. N., Niebergall L., Longo R. C. & Bruno P. Adatom self-organization induced by quantum confinement of surface electrons. Phys. Rev. Lett. 97, 186403 (2006). 17155563 10.1103/PhysRevLett.97.186403

[b31] Cheng Z. *et al.* Adsorbates in a box: Titration of substrate electronic states. Phys. Rev. Lett. 105, 066104 (2010). 20867991 10.1103/PhysRevLett.105.066104

[b32] Cao R. X. *et al.* Two-dimensional quantum diffusion of Gd adatoms in nano-size Fe corrals. Phys. Rev. B 87, 085415 (2013).

[b33] Cao R. X. *et al.* Self-regulated Gd atom trapping in open Fe nanocorrals. Phys. Rev. B 90, 045433 (2014).

[b34] Li J., Schneider W.-D. & Berndt R. Local density of states from spectroscopic scanning-tunneling-microscope images: Ag(111). Phys. Rev. B 56, 7656–7659 (1997).

[b35] Hu J. *et al.* Size-dependent quantum diffusion of Gd atoms within Fe nano-corrals. Surf. Sci. 618, 148–153 (2013).

[b36] Ding H. F. *et al.* Electron-beam tip/sample heating device for a scanning tunneling microscopy. Rev. Sci. Instrum. 76, 123703 (2005).

[b37] Ternes M. *et al.* Scanning-tunneling spectroscopy of surface-state electrons scattered by a slightly disordered two-dimensional dilute “solid”: Ce on Ag(111). Phys. Rev. Lett. 93, 146805 (2004). 15524828 10.1103/PhysRevLett.93.146805

[b38] Cao R. X. *et al.* Spectroscopy of self-assembled one-dimensional atomic string: The role of step edge. Appl. Phys. Lett. 103, 081608 (2013).

