# Supplementary material for: Spectroscopic study of Gd nanostructures quantum confined in Fe corrals
Source: Sci Rep. 2015 Jul 10;5:12092. doi: 10.1038/srep12092 (PMC4498218; doi:10.1038/srep12092)
Supplement: Supplementary Information [file srep12092-s1.pdf]

Supplementary Information

Spectroscopic study of Gd nanostructures quantum confined in Fe corrals

R. X. Cao[1], L. Sun[1,2], B. F. Miao[1], Q. L. Li[1], C. Zheng[1], D. Wu[1,2], B. You[1,2], W. Zhang[1,2], P. Han[2,3], S. D. Bader[4], W. Y. Zhang[1,2], and H. F. Ding[1,2*]

[1]*National Laboratory of Solid State Microstructures and Department of Physics, Nanjing University, 22 Hankou Road, Nanjing 210093, People's Republic of China*

[2]*Collaborative Innovation Center of Advanced Microstructures, Nanjing University, 22 Hankou Road, Nanjing 210093, P. R. China*

[3]*School of Electronic Science and Engineering, Nanjing University, 22 Hankou Road, Nanjing 210093, People's Republic of China*

[4]*Materials Science Division, Argonne National Laboratory, Argonne, Illinois 60439, USA*

*Corresponding author: hfding@nju.edu.cn

1. Quantum confinement of Gd structures

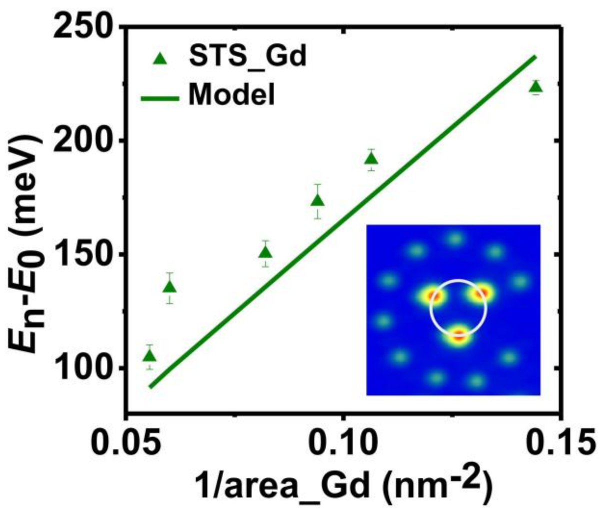

Supplementary Figure S1. Comparison of spectroscopic peak positions derived from experiments for six different-sized Gd-filled corrals (green triangles) and the quantum-confinement model of the Gd structures (green line). The effective area of the Gd structures was calculated by the white circle marked in the inset.

The energy levels were calculated utilizing the quantum-confinement model $E_n = E_0 + \frac{\hbar^2 z_n^2}{2m^* r^2}$ mentioned in the text. The effective radius was calculated by the circular orbit occupied by the Gd adatoms, which is marked as a white circle in the inset of Fig. S1. The calculated results for different-sized, circular Gd structures are shown as the green line in Fig. S1. For comparison, the experimental spectroscopic peak positions are plotted as green triangles in Fig. S1. Qualitative agreement is obtained, as in Fig. 2 in the text.

2. Tight-binding calculation for the 8.5-nm corral

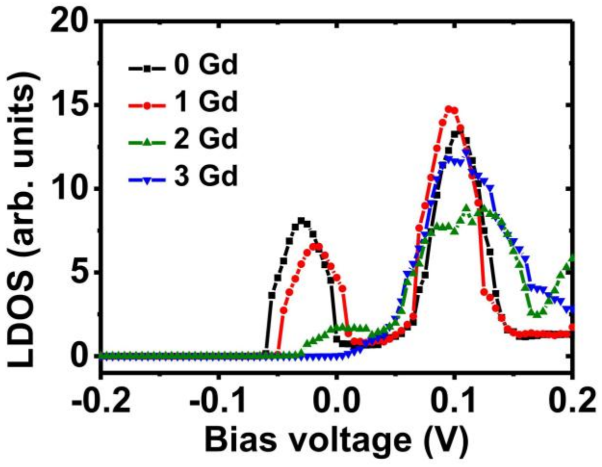

Supplementary Figure S2. The change of the tight-binding calculated local density of states at the center of the 8.5-nm corral during trapping Gd adatoms.

Utilizing the tight-binding calculations, the step–by-step evolution of local density of states in the 8.5-nm corral was investigated as it traps Gd adatoms (Fig. S2). The intensity of the peak at lower energy decreases to zero gradually and its position shifts slightly towards higher energy; the intensity and position of the peak at higher

energy are similar, except for the case of trapping of two Gd adatoms. The calculated results agree with the experiments shown in Fig. 4.

3. Comparison of positions of corral state's nodes and interaction derived Gd adatoms

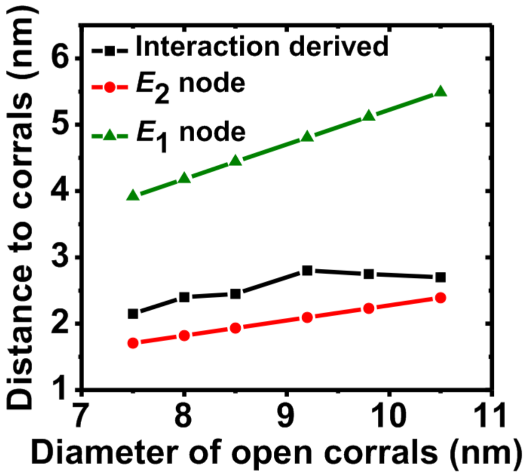

Supplementary Figure S3. Comparison of the positions of long-range interaction energy minimum (black rectangles), the first (green triangles) and second (red circles) corral state's nodes in different-sized corrals.

The calculated distances from Gd adatoms to Fe open corrals (black rectangles) were derived from the energy minimum similar to Fig. 3(a) in the text. The positions of corral state's nodes were calculated from the combination of quantum confined energy levels $E_n = E_0 + \frac{\hbar^2 z_n^2}{2m^* r^2}$ and dispersion relationship $E = E_0 + \frac{\hbar^2 k^2}{2m^*}$. We note that the phase shift of scattering by Fe adatom $\sim 0.4\pi$ was taken into consideration [Phys. Rev. B **87**, 085415, (2013)]. We can find that the Gd position is close to the

node of E_2 but far away from that of E_1. In such case, the wavefunction (spectrum) of E_2 almost remains unchanged while that of E_1 vanishes after the trapping of Gd adatoms.
